# Exosomes enriched by miR-429-3p derived from ITGB1 modified Telocytes alleviates hypoxia-induced pulmonary arterial hypertension through regulating Rac1 expression

**DOI:** 10.1007/s10565-024-09879-0

**Published:** 2024-05-20

**Authors:** Ruixue Qi, Yong Zhang, Furong Yan

**Affiliations:** 1 Center for Tumor Diagnosis and Therapy, Jinshan Hospital, Fudan University, Shanghai, China; 2 Department of Respiratory Medicine, Zhongshan Hospital, Fudan University, Shanghai, China

**Keywords:** ITGB1, miR-429-3p, Exosomes, Rac1, PAH, Inflammation

## Abstract

**Background:**

Recent studies have emphasized the critical role of Telocytes (TCs)-derived exosomes in organ tissue injury and repair. Our previous research showed a significant increase in ITGB1 within TCs. Pulmonary Arterial Hypertension (PAH) is marked by a loss of microvessel regeneration and progressive vascular remodeling. This study aims to investigate whether exosomes derived from ITGB1-modified TCs (ITGB1-Exo) could mitigate PAH.

**Methods:**

We analyzed differentially expressed microRNAs (DEmiRs) in TCs using Affymetrix Genechip miRNA 4.0 arrays. Exosomes isolated from TC culture supernatants were verified through transmission electron microscopy and Nanoparticle Tracking Analysis. The impact of miR-429-3p-enriched exosomes (Exo-ITGB1) on hypoxia-induced pulmonary arterial smooth muscle cells (PASMCs) was evaluated using CCK-8, transwell assay, and inflammatory factor analysis. A four-week hypoxia-induced mouse model of PAH was constructed, and H&E staining, along with Immunofluorescence staining, were employed to assess PAH progression.

**Results:**

Forty-five miRNAs exhibited significant differential expression in TCs following ITGB1 knockdown. Mus-miR-429-3p, significantly upregulated in ITGB1-overexpressing TCs and in ITGB1-modified TC-derived exosomes, was selected for further investigation. Exo-ITGB1 notably inhibited the migration, proliferation, and inflammation of PASMCs by targeting Rac1. Overexpressing Rac1 partly counteracted Exo-ITGB1’s effects. In vivo administration of Exo-ITGB1 effectively reduced pulmonary vascular remodeling and inflammation.

**Conclusions:**

Our findings reveal that ITGB1-modified TC-derived exosomes exert anti-inflammatory effects and reverse vascular remodeling through the miR-429-3p/Rac1 axis. This provides potential therapeutic strategies for PAH treatment.

**Graphical Abstract:**

1. Identification of Differentially Expressed microRNAs (DEmiRs) in ITGB1 overexpressed TCs.

2. Effects of Exo-ITGB1 or miR-429-3p on Hypoxia-Induced PASMCs in vitro.

3. Exo-ITGB1 inhibits the hyper-proliferation and migration of PASMCs through regulating miR-429-3p/Rac1 axis in vitro.

4. The therapeutic potential of Exo-ITGB1 in hypoxia-induced PAH model in vivo.

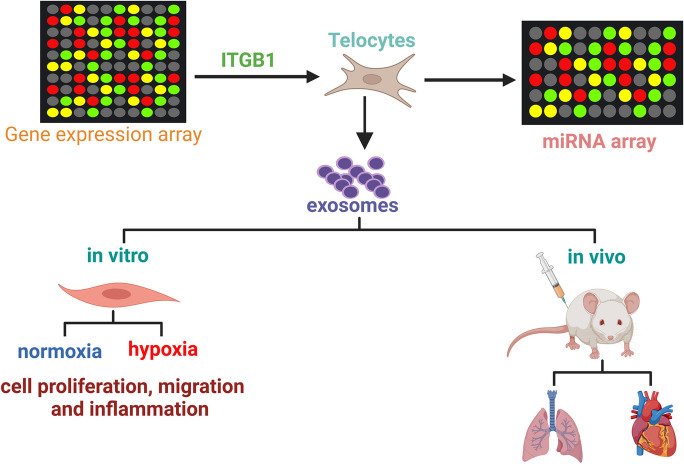

**Supplementary Information:**

The online version contains supplementary material available at 10.1007/s10565-024-09879-0.

## Introduction

Pulmonary Arterial Hypertension (PAH), characterized by elevated pulmonary pressure and extensive vascular remodeling, is a chronic, progressive disease, potentially leading to heart failure and death(Schermuly et al. 2011; Thenappan et al. 2018a; Kang et al. 2022). Its pathogenesis involves complex interplays of cellular and molecular mechanisms, including endothelial dysfunction, vasoconstriction, thrombosis, inflammation, and vascular remodeling(Grinnan et al. 2019). Vascular remodeling impacts various layers of the pulmonary vascular wall, comprising fibroblasts, endothelial cells, and PASMCs(Riou et al. 2023). While the exact cause of PAH remains elusive, it is considered a combination of environmental and genetic factors(Ruopp and Cockrill 2022). Current treatments primarily focus on vasodilation by targeting phosphodiesterase, prostacyclin, and endothelin-1 receptors(Humbert et al. 2022). While these approaches improve quality of life and alleviate symptoms, they do not effectively halt disease progression, even with novel combinations of multiple drugs (Ruopp and Cockrill 2022). Therefore, it is crucial to explore and understand novel cellular processes and their underlying mechanisms in order to develop innovative approaches for the treatment of this disease.

Telocytes (TCs) are unique stromal cells with long extensions called telopodes (Tps), featuring podomers and podoms (Cretoiu and Popescu 2014; Faussone and Popescu 2011). TCs were found to be observed in a number of tissues and organs, including the skin, heart, spleen, lungs, and female reproductive system over the last decade (Zhang et al. 2020; Cretoiu et al. 2017; Cretoiu and Xu 2016). Increasing evidence have shown that TCs performed multiple biological functions and participated in extensive crosstalk with neighboring tissues and cells due to special structure (Popescu et al. 2016; Sukhacheva et al. 2021). Our previous studies demonstrated that TCs from mouse lung distinguished from other local cell types, including MSCs, airway basal cells, alveolar type II cells, proximal airway cells, fibroblasts, CD8( +) T cells from bronchial lymph nodes and lungs (Song et al. 2016; Wang et al. 2015). Following experiments have constructed mouse lung TCs which maintained the biological properties and behaviors as primary TCs (Song et al. 2019). ITGB1, an integrin subunit essential for extracellular matrix interactions and several signaling channels, such as Hippo pathway, Wnt/β-catenin pathways and Fak signaling (Liu et al. 2010; Chang et al. 2021; Li et al. 2021). ITGB1 was linked to inflammatory reactions in injuries resulting from renal ischemia and reperfusion (Chen et al. 2020), suggesting its role in protecting pulmonary arteries via cell–cell and cell-ECM interactions.

In this study, we overexpressed ITGB1 in TCs to explore its role in tissue repair. We identified differentially expressed microRNAs (miRNAs) and found that miR-429-3p, decreased in ITGB1 knockdown TCs and ITGB1-modified TC-derived exosomes, played a significant role in vascular remodeling. Our experiments aimed to clarify the function and significance of Exo-ITGB1 in vascular remodeling, thereby exploring the potential of exosomes from ITGB1-modified TCs in a hypoxia-induced PAH model.

## Methods

### Cell isolation and culture

Telocytes (TCs) from mouse lungs and PASMCs from C57BL/6 mice were cultivated in DMEM with FBS and antibiotics as previously described (Zhang et al. 2020). They were kept in the cell incubators containing 5% carbon dioxide at 37 °C. For hypoxia studies, cells underwent 48 h in a 5% O^2^ environment.

### RNA isolation and qRT-PCR

Exosome RNA was isolated using an Exosome RNA Purification Kit. For miR-429-3p expression analysis, using TaqMan MicroRNA Reverse Transcription Kit, 100 ng of RNA was reverse-transcribed, after amplified, then specific probed using the Real-time PCR System Applied Biosystems 7500.

### MiRNA microarray analysis

MiRNAs profiling in TCs was conducted using the Affymetrix GeneChip® miRNA 4.0 Array. This involved RNA isolation, Poly(A) tailing, and flashtag biotin HSR ligation, followed by hybridization, washing, and scanning. Differentially expressed miRNAs were identified based on fold change and statistical significance.

### Bioinformatic analysis

The adhesion-related feature gene was gathered from Mouse Molecular Signatures Database (MSigDB, v2023.1, https://www.gsea-msigdb.org/gsea/msigdb/index.jsp) (Liberzon et al. 2015) with the keywords: adhesion gene.

The potential targets of miR-429-3p were predicted through publicly available different miRNA databases such as TargetScan (http://www.targetscan.org/), miRDB (https://mirdb.org/) and ENCORI (https://starbase.sysu.edu.cn/).

### Isolation and characterization of exosomes

Exo-Ve and Exo-ITGB1, respectively, are the names given to exosomes that were separated from TCs or ITGB1 overexpressed TCs. Thermofisher’s Total Exosome Isolation Reagent was used to extract exosomes from cell culture media in accordance with the instructions. As previously mentioned, TEM, western blotting, and nanoparticle tracking analysis were used to characterize exosomes(Zhang et al. 2020).

### Building plasmids and transfecting cells

PCR was used to amplify the full-length cDNA of the ITGB1 gene from TCs. The pCDH-CMV-MCS-EF1-Puro lentiviral vector (SBI, USA) was cloned containing the PCR results. Sequencing was used to identify the plasmids. ITGB1-carrying lentiviruses were generated using co-transfection of pCDH-ITGB1, pMD2.G, and pSPAX.HEK293T cells were transfected with two plasmids using Lipofectamine 3000 (Invitrogen). After 48 h, the viral supernatant was extracted and combined with 4 μg/mL polybrene (sourced from Sigma-Aldrich) to be transduced into TCs. For 48 h, TCs were sorted using 2 μg/mL puromycin.

To enforce Rac1 expression, the plasmid containing the coding sequence of Rac1 was cloned into pcDNA3.1 (Addgene plasmid: V790-20) and transfected into PASMCs using Lipofectamine 3000 (Thermo-Fisher) according to the instructions of the kits.

### CCK-8 assay and EdU

PASMC viability was assessed using the CCK-8 assay. The proliferation was measured using the EdU assay, with cells incubated in EdU, fixed and stained with a Click solution and DAPI. The proliferation rate was calculated based on EdU-positive cells.

### Wound closure and invasion assays

For wound healing assays, PASMCs were grown to confluence, wounded, and imaged at 0 and 24 h. Migration was assessed using a Transwell setup, with PASMCs migrating towards a DMEM-FBS gradient, fixed, and stained for analysis.

### Animal model

The national criteria for the care and use of animals were followed when conducting animal experiments. Ethical approval was acquired from Institutional Animal Care and Use Committee of SHZY (Permit No. SHZY-20220316X). A PHA model was performed as described before (Omura et al. 2019). In brief, mice were randomly divided into four groups: mice treated with PBS (VV), mice treated with hypoxia group (10%O_2_ and 90% N_2_), mice treated with hypoxia (10%O_2_ and 90%N_2_) and Exo-Ve (2 μg/kg body weight), mice treated with hypoxia (10%O_2_ and 90%N_2_) and Exo-ITGB1 (2 μg/kg body weight) every week. The oxygen concentration was controlled by the ProOx-100HE chamber. All mics were terminated by the overdose of pentobarbital.

### Immunofluorescence staining

The immunofluorescence staining for Rac1 in PASMCs involved fixing, blocking, and incubating with primary and secondary antibodies, followed by DAPI staining. CD34, Vimentin, and PDGFα was performed as our previously reported (Wang et al. 2020). Fluorescence images were captured with the fluorescence microscope.

### Enzyme-linked immunosorbent assay

Using particular mice ELISA kits, the levels of IL-10, IL-6, and IL-1β in bronchoalveolar lavage fluid (BALF) were measured (Invitrogen), following protocols provided by the manufacturer.

### Histological analysis

The pulmonary artery morphology was analyzed via Hematoxylin and Eosin (H&E) staining and Masson’s trichrome staining. The right ventricular hypertrophy morphology was conducted using (H&E) staining. These procedures were applied to lung and heart tissues obtained post-euthanasia, with the techniques following established protocols(Chen et al. 2017). We quantify remodeling by measuring the percentage of inner wall thickness of pulmonary vascular vessels.

### Hemodynamic evaluation

Hemodynamic parameters were measured using a protocol involving anesthesia with pentobarbital and catheterization of the right external jugular vein at the end of the fourth week of hypoxia exposure as described previously (Omura et al. 2019). Then a catheter was inserted into the right ventricle (RV), and the pressure waveform was continuously monitored in real-time. The right ventricular systolic pressure (RVSP) was recorded over a 10-min period using the ML866 Power Lab Data Acquisition Systems.

### Dual luciferase reporter assay

The wild-type 3' UTR of the Rac1 gene (Rac1-WT) was cloned into psiCHECK-2 vector, while a mutated version of the Rac1 (Rac1-MUT) was generated using Site-Directed Mutagenesis Kits (Agilent). HEK293 cells were co-transfected with 200 ng of either Rac1-WT or Rac1-MUT and 50 ng of Exo-Ve or Exo-ITGB1 using Lipofectamine™ 3000. In each sample of the Dual-Luciferase Assay Kits, the Luciferase activity was subsequently measured according to the instructions.

### Analysis of the western blot

Protease/phosphatase inhibitors were added to RIPA buffer during the preparation of cell lysates. Using a BCA kit, protein concentrations were measured. After that, equal volumes of protein were separated using SDS-PAGE and put on PVDF membranes. Following blocking, primary antibodies against ITGB1, Rac1, and GAPDH were incubated on the membranes. The Pierce™ ECL Plus Western Blotting Substrate (Thermo Scientific™) was used for detection, and ImageJ was used to quantify band intensities.

### Statistical analysis

The three separate experiments' means and standard deviations are shown in the data. A paired or unpaired Student's t-test was used for two-group comparisons, and a one-way ANOVA and Tukey's post hoc test were used for multiple group comparisons. To do statistical analysis, GraphPad Prism (version 5.0) was used. Every experiment was conducted three times, with a significance level of P < 0.05.

## Results

### Overexpression of ITGB1 in TCs

**Fig. 1:**
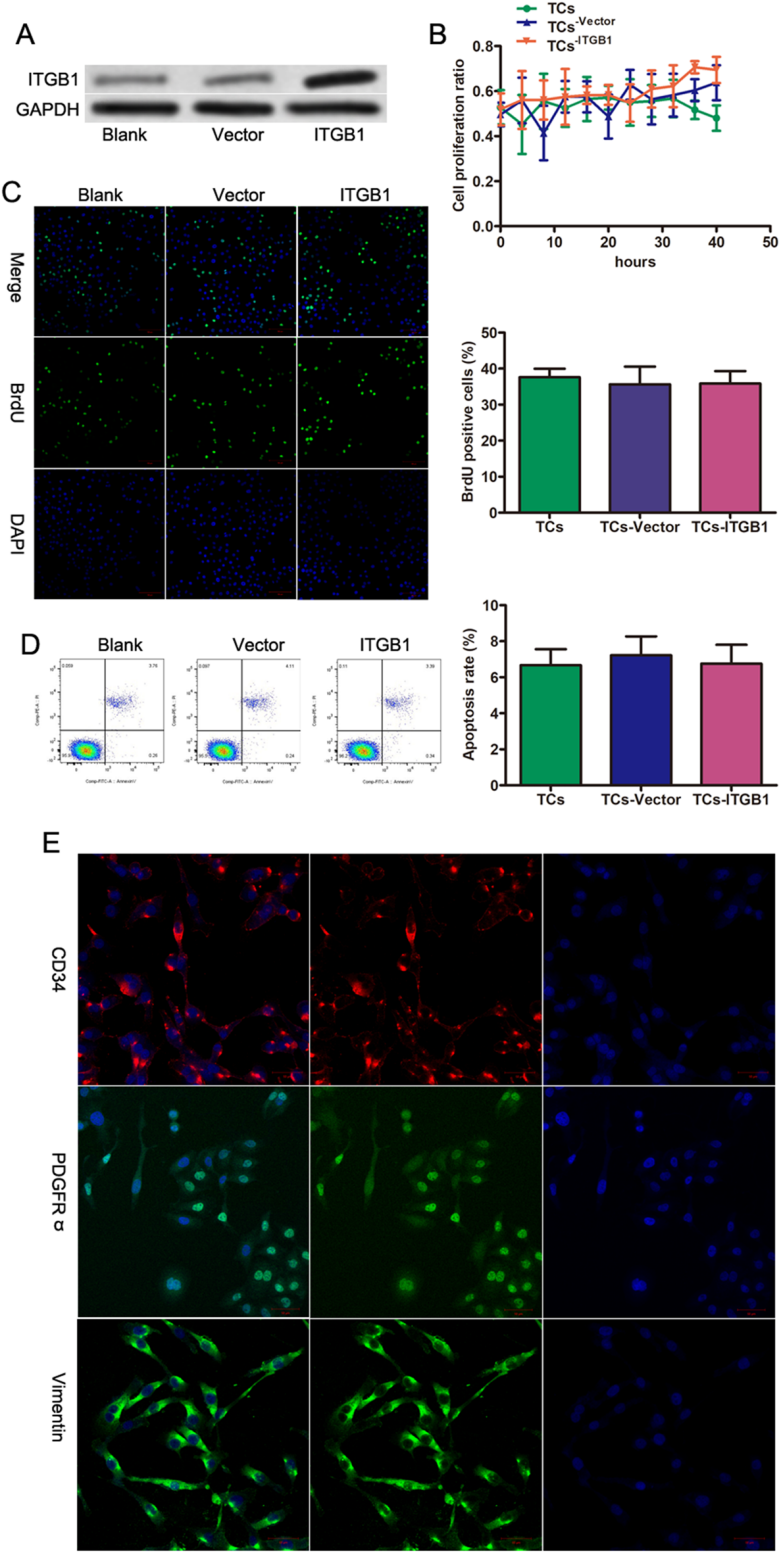
The effects of ITGB1 on TCs. (**A**) ITGB1 protein level was determined in TCs after ITGB1 overexpression using immunoblotting, GAPDH served as a loading control. Cell proliferation analysis (**B**) and EdU incorporation assay (**C**) of TCs transfected with or without ITGB1. (**D**) Flow cytometry analysis showed that apoptosis level of TCs transfected with or without ITGB1. (**E**) Immunofluorescence labelling for CD34, PDGFRα and vimentin with DAPI counterstain for nuclei

ITGB1 is highly expressed in mouse lung TCs, which is involved in tissue healing and repair, according to our earlier studies. To examine ITGB1's impact on TCs, it was overexpressed in TCs (Fig. 1A). CCK-8 (Fig. 1B) and EdU assays (Fig. 1C) showed no significant changes in TCs post-ITGB1 transfection. Flow cytometry revealed no notable apoptosis alteration (Fig. 1D). Immunofluorescent staining for CD34, PDGFRα, and vimentin indicated that ITGB1 overexpression did not affect TCs' characteristic markers (Fig. 1E), suggesting ITGB1 overexpression does not significantly impact TCs' biological functions.

### Identification of DEmiRs

**Fig. 2:**
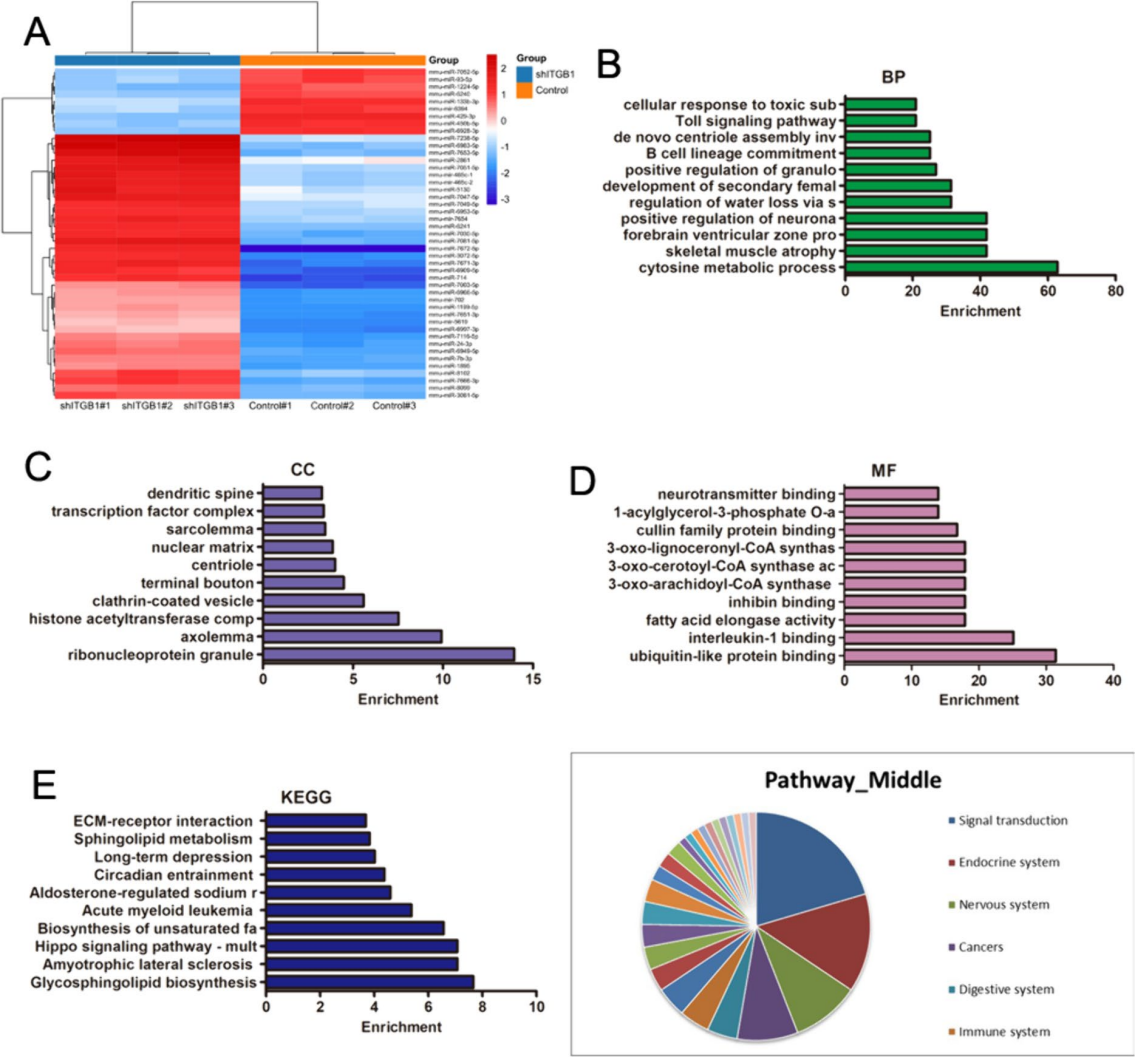
Identification of differentially expressed miRNAs and functional enrichment. (**A**) Heatmap of miRNAs via hierarchical cluster analysis. Colors from green to red represented the abundance from poor to rich. (**B-D**) Histograms show gene ontology (GO) analysis of biological processes enriched (**B**), cellular component (**C**) and molecular function (D) for the down-regulated DEmiRs after ITGB1 knockdown. (**E**) KEGG pathways enrichment for the down-regulated DEmiRs after ITGB1 knockdown. (**F**) Pie chart of KEGG

An essential function of integrin subunit beta 1 (ITGB1) is to facilitate tissue repair processes (Klingler et al. 2021; Rai et al. 2021). TCs participate in intercellular communication and signal networks via extracellular vesicle release (Cismasiu and Popescu 2015; Fertig et al. 2014). Exosome miRNAs are stabilized stored, and some miRNAs are higher in exosomes than in their parent cells (Thomou et al. 2017; Asgarpour et al. 2020). Hence, miRNA profiling was conducted, identifying 45 DEmiRs with twofold change and P < 0.05 (Table 1). Heat maps displayed 36 upregulated and 9 downregulated miRNAs (Fig. 2A). A qRT-PCR assay further confirmed the decreased expression of mmu-miR-429-3p, mmu-miR-450b-5p, mmu-miR-6928-3p, mmu-miR-7052-5p, mmu-miR-1224-5p, mmu-miR-133b-3p, mmu-miR-6240, mmu-mir-6394, and mmu-miR-93-5p in ITGB1-associated exosomes (Figure S1). Gene ontology analysis was conducted over the pool of significantly downregulated DEmiRs in ITGB1-depleted TCs. GO terms for biological processes (BP) were enriched in cytosine metabolic process, skeletal muscle atrophy and forebrain ventricular zone progenitor cell division (Fig. 2B). GO terms for cellular component (CC) were enriched in ribonucleoprotein granule, axolemma histone acetyltransferase complex and clathrin-coated vesicle (Fig. 2C). GO terms for cellular component (CC) were enriched in ubiquitin-like protein binding, interleukin-1 binding and fatty acid elongase activity (Fig. 2D). KEGG pathway analysis from downregulated DEmiRs after ITGB1 depletion showed enrichment in Glycosphingolipid biosynthesis-globo and isoglobo series, Amyotrophic lateral sclerosis (ALS) and Hippo signaling pathway-multiple species (Fig. 2E). The pie chat shows overall contribution of the pathways and signal transduction has the highest contribution. On the other hand, analysis of upregulated DEmiRs after ITGB1 knockdown showed enrichment in GO terms and pathways associated with negative regulation of smoothened signaling pathway involved in dorsal/ventral neural tube patterns, ubiquitin-like protein binding, and Hedgehog signaling pathway (Figure S2).

**Table 1:** Detail of alteration of miRNAs in TCs after ITGB1 knockdown

Gene	Fold change	Gene feature
mmu-miR-429-3p	-2.585	down
mmu-miR-450b-5p	-2.25	down
mmu-miR-6928-3p	-2.235	down
mmu-miR-7052-5p	-2.014	down
mmu-miR-1224-5p	-2	down
mmu-miR-133b-3p	-2	down
mmu-miR-6240	-2	down
mmu-mir-6394	-2	down
mmu-miR-93-5p	-2	down
mmu-miR-8102	2	up
mmu-mir-5619	2.014	up
mmu-miR-6966-5p	2.014	up
mmu-miR-6997-3p	2.014	up
mmu-mir-702	2.014	up
mmu-miR-7651-3p	2.014	up
mmu-miR-7b-3p	2.014	up
mmu-miR-8099	2.014	up
mmu-miR-1895	2.085	up
mmu-miR-1199-5p	2.099	up
mmu-miR-7049-5p	2.114	up
mmu-miR-6953-5p	2.129	up
mmu-miR-2861	2.173	up
mmu-miR-6949-5p	2.173	up
mmu-miR-7116-5p	2.219	up
mmu-miR-24-3p	2.235	up
mmu-miR-3081-5p	2.25	up
mmu-miR-5130	2.282	up
mmu-miR-7047-5p	2.282	up
mmu-mir-7654	2.395	up
mmu-miR-6241	2.532	up
mmu-miR-7666-3p	2.532	up
mmu-mir-465c-1	2.657	up
mmu-mir-465c-2	2.657	up
mmu-miR-7051-5p	2.657	up
mmu-miR-7003-5p	2.732	up
mmu-miR-7030-5p	2.732	up
mmu-miR-3072-5p	3.095	up
mmu-miR-7081-5p	3.117	up
mmu-miR-7238-5p	3.16	up
mmu-miR-6909-5p	3.411	up
mmu-miR-6983-5p	3.482	up
mmu-miR-7653-5p	3.482	up
mmu-miR-7671-3p	3.482	up
mmu-miR-714	3.655	up
mmu-miR-7672-5p	4.532	up

### miR-429-3p in ITGB1-modified Telocytes-derived exosomes

**Fig. 3:**
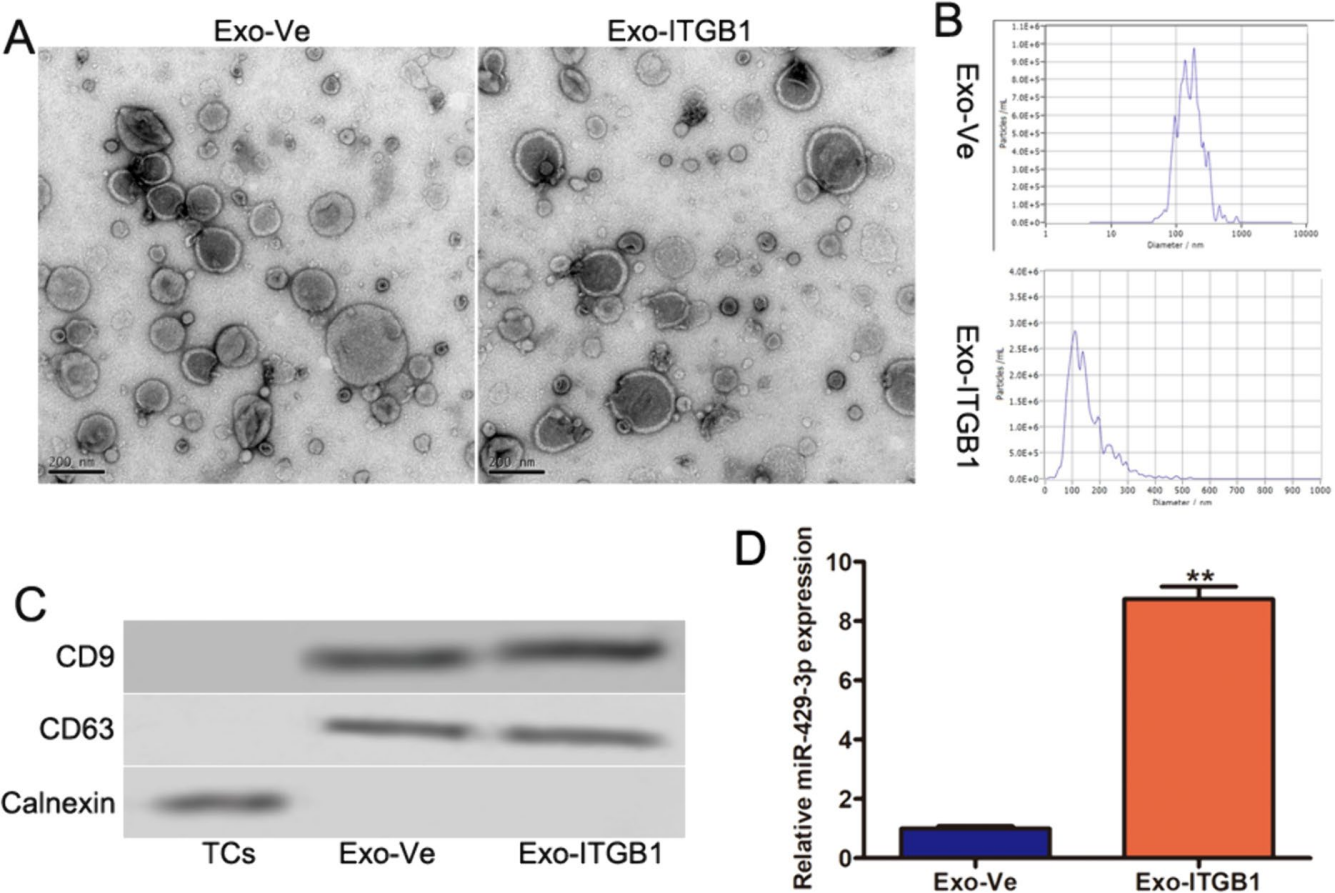
Characterization of exosomes derived from TCs. (**A**) Micrographs of exosomes by TEM. Scale bar, 200 nm. (**B**) Particle size of exosomes by Nanoparticle Tracking Analysis (NTA). (**C**) Western blot analysis of CD9, CD63 and expression in exosomes. (**D**) The expression of miR-429-3p in TCs-Vector and TCs-ITGB1 by real-time PCR. **p < 0.01

The encapsulation of miRNAs within exosomes provides protection against degradation by RNases and enhances their stability in circulation. Moreover, exosomes serve as vehicles for selective delivery of miRNAs to target cells, increasing their efficiency and specificity (Asgarpour et al. 2020). Exosomes showed characteristic features under transmission electron micrographs (TEM) (Fig. 3A) and had a particle size of 50–200 nm per NTA data (Fig. 3B). Western blot confirmed the presence of CD9 and CD63 and absence of calnexin in exosomes (Fig. 3C). The above downregulated miRNAs were further detected in exosomes. Among downregulated miRNAs, miR-429-3p was highly expressed post-ITGB1 overexpression (Fig. 3D), indicating its key role in Exo-ITGB1's biological functions.

### Effects of Exo-ITGB1 on hypoxia-induced PASMCs

**Fig. 4:**
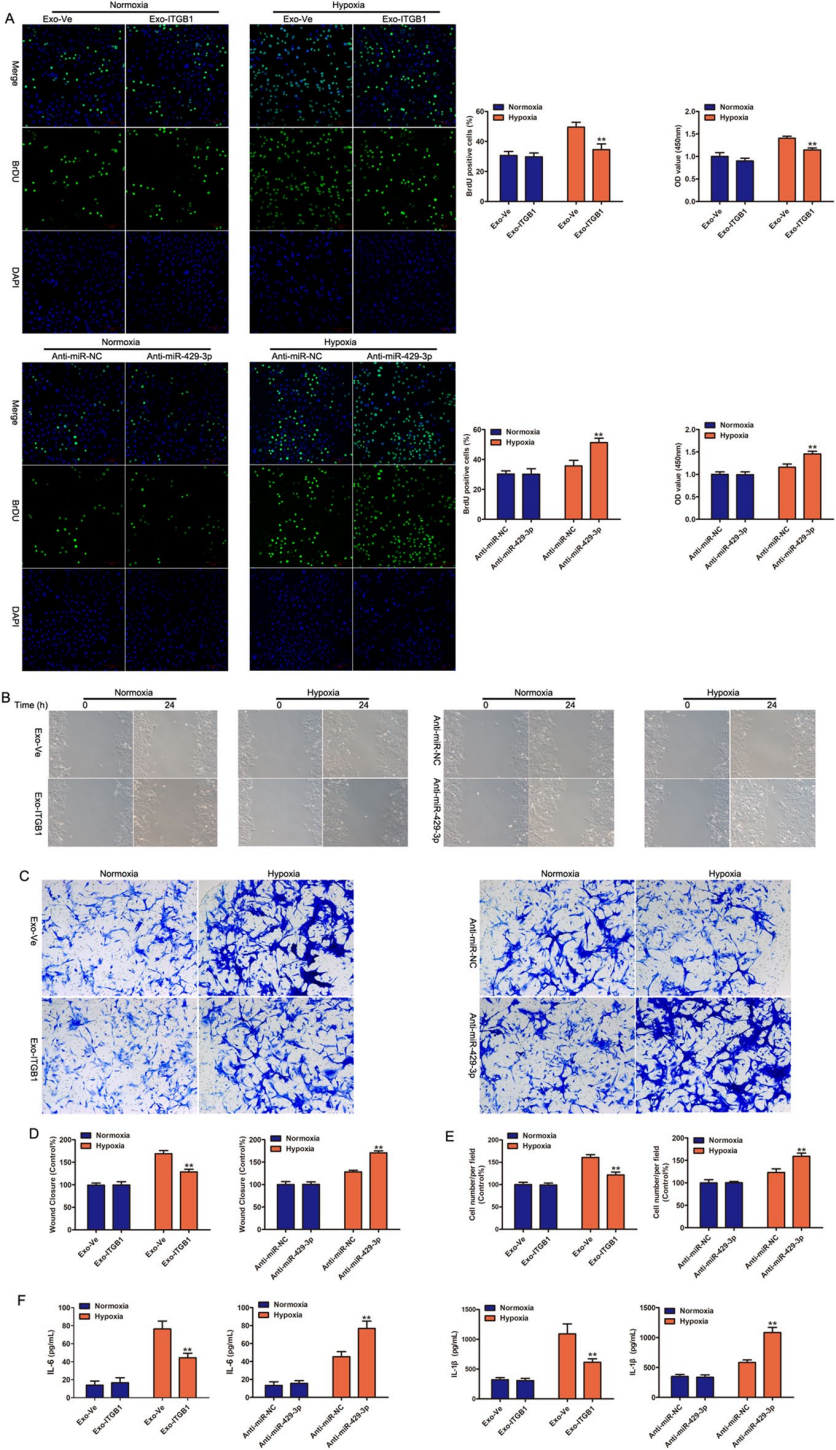
Effect of Exo-ITGB1 on hypoxia-induced PASMCs proliferation, migration and inflammation. PASMCs was treated with Exo-Ve or Exo-ITGB1, Anti-NC or Anti-miR-429-3p under the basal and hypoxia condition (2% O_2_, 5% CO_2_, and 93% N_2_). (**A**) EdU incorporation and CCK- 8 assay were used to assess the cell viability and quantitative data was presented as mean ± standard deviation (SD). (**B** and **C**) Scratch wound assay (**B**) and Transwell assay (**C**) were performed to measure the migrative ability of PASMCs. (**D** and **E**) Quantitation of B (**D**) and (**E**) from three independent experiments. (**F**) The content of IL-6 and IL-1β in cell supernatant. **p < 0.01 versus normal; #*p* < 0.05, Exo-Ve vs. Exo-ITGB1

In a study looking at how Exo-ITGB1 affects alterations brought on Pulmonary Arterial Smooth Muscle Cells (PASMCs), significant findings emerged. Under hypoxic conditions, Exo-ITGB1 notably reduced the proliferation of PASMCs, as demonstrated by EdU staining and CCK8 assays, whereas such changes were not observed under normoxic conditions. Interestingly, the inhibition of miR-429-3p led to an increase in PASMC proliferation under hypoxia but had no effect under normoxia (Fig. 4A). Furthermore, hypoxia stimulation significantly enhanced migration ability, while Exo-ITGB1 treatment significantly reversed the effect of hypoxia stimulation. No significant difference was observed between them under normoxia condition (Fig. 4B-E). Furthermore, inflammatory factors such as IL-6 and IL-1βremarkably increased after hypoxia exposure, while Exo-ITGB1 alleviated the effect remarkably (Fig. 4F). These data together proved that Exo-ITGB1 might attenuate pulmonary vascular remodeling of PAH by suppressing proliferation and migration of PASMCs.

### miR-429-3p regulates Rac1 expression

**Fig. 5:**
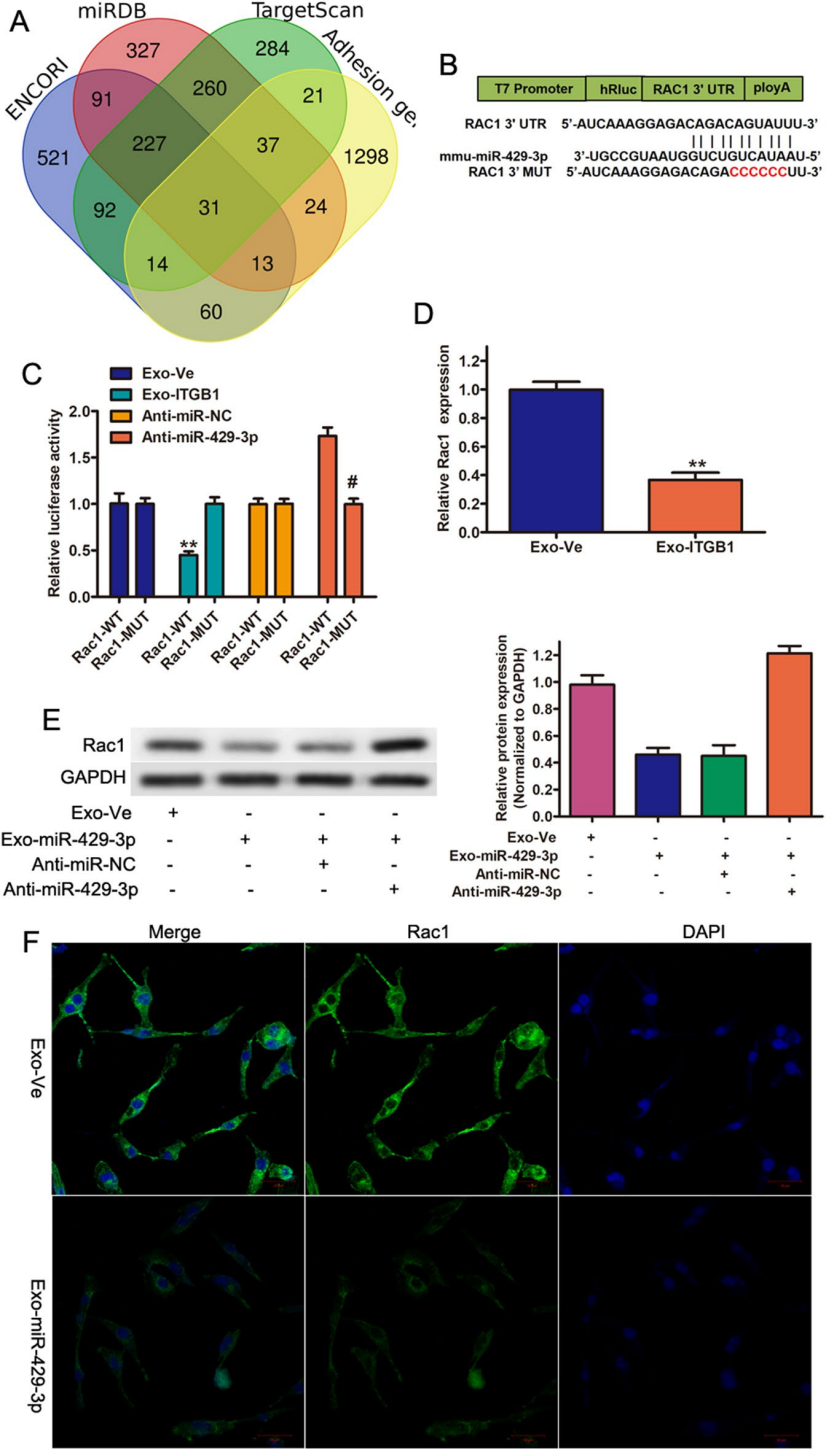
miR-429-3p targets and negatively regulates Rac1. (**A**) Venn diagram of potential targets of miR-429-3p from Starbase, miRDB, TargetScan and adhesion-related genes. (**B**) The complementary binding site between Rac1 and miR-429-3p was predicted by TargetScan database. (**C**) The direct interaction between miR-429-3p and Rac1 was confirmed by a dual luciferase reporter gene assay. (**D**) Rac1 expressions in PASMCs following Exo-ITGB1 treatment were assessed using qRT-PCR. (**E**) The protein level of Rac1 in PASMCs following Exo-ITGB1 treatment with or without inhibitors of miR-429-3p transfection. (**F**) Immunofluorescence images of the Rac1 expression in PASMCs with or without the presence of Exo-ITGB1. *p < 0.05; **p < 0.01

It has been illustrated that miR-429 exerts its functions by targeting multiple genes, such as Fasciculation And Elongation Protein Zeta 2, Angiotensin Converting Enzyme 2, MYC, BCL2, and EGFR (Meng et al. 2023; Gheidari et al. 2022; Zhang et al. 2023).To further explore the functional role of miR-429-3p in pulmonary vascular remodeling, bioinformatics workflow was used to identify putative target genes of miR-429-3p. As illustrated in Fig. 5A, a total of 31 genes were predicted as the potential targets of miR-429-3p based on online database for miRNA target prediction. Rac1 was choose for further experiments due to RAC1 activation leads to increased proliferation of PASMCs and contributes to the development of pulmonary vascular remodeling (Fig. 5B) (Zheng et al. 2023; Jin et al. 2022; Dilasser et al. 2022). The reports results of dual luciferase showed that compared to the Exo-Ve, the luciferase activity of Rac1-WT showed significant inhibition of by Exo-ITGB1, while no difference was observed in the Rac1-MUT (Fig. 5C). In contrast, the impacts of Exo-ITGB1 on the luciferase activity of Rac1-WT were significantly blocked by inhibitors of miR-429-3p (Anti-miR-429-3p) (Fig. 5C). Furthermore, the protein and mRNA expression of Rac1 dramatically decreased in PASMCs after Exo-ITGB1 treatment (Fig. 5D and E). Interestingly, Anti-miR-429-3p could reversed the inhibition of Rac1 by Exo-ITGB1 (Fig. 5E). In addition, it was showed that Rac1 level in PASMCs was markedly suppressed after Exo-ITGB1 treatment by immunofluorescence (Fig. 5F). Taken together, it was suggested that miR-429-3p suppressed Rac1 expression in PASMCs through a direct binding relationship.

### Rac1's role in Exo-ITGB1's effects on PASMCs

**Fig. 6:**
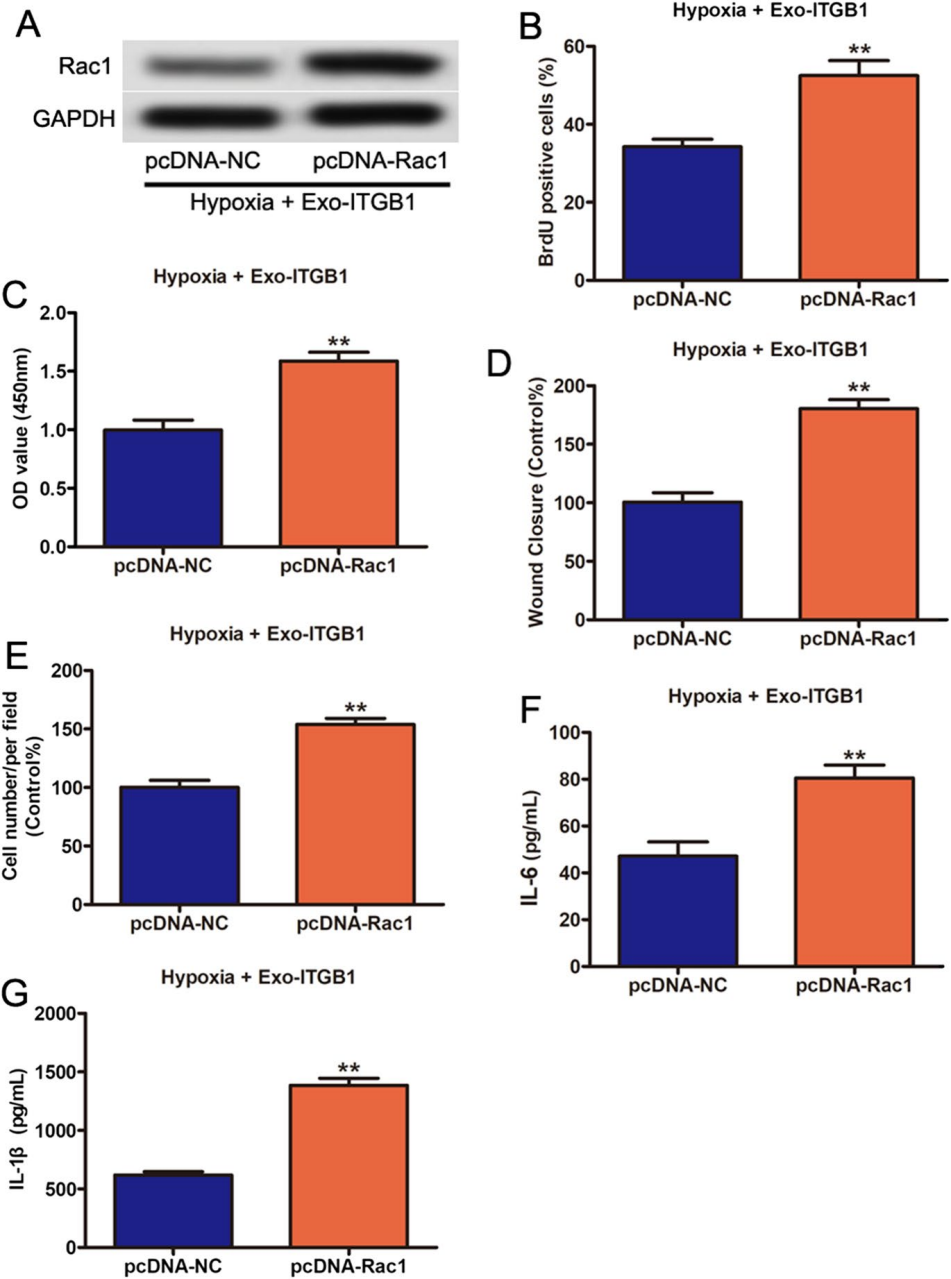
Overexpression of Rac1 partly reverses the inhibition effects of Exo-ITGB1 on PASMCs. PASMCs were treated with Exo-ITGB1 after hypoxia. Then cells were transfected pcDNA-NC or pcDNA-ITGB1. (**A**) The transfection efficiency of Rac1 was validated using western blotting. (**B** and **C**) EdU incorporation and CCK-8 assays were used to detect cell viability. (**D** and **E**) Scratch wound assay and Transwell assay were performed to assess the migrative ability of PASMCs. (**F** and **G**) The content of IL-6 and IL-1β in cell supernatant as indicated treatment. *p < 0.05; **p < 0.01

Then, we explored Rac1 was involved in the biological effects of Exo-ITGB1, ectopic overexpression of Rac1 in PASMCs treated with Exo-ITGB1, which was verified with Western blot (Fig. 6A). Ectopic overexpression of Rac1 synergistically attenuated the inhibition effects of Exo-ITGB1 on cell viability of PASMCs (Figs. 6B and C). The data of Scratch wound assay (D) and Transwell assay (E) also showed that the decrease of migrative ability by Exo-ITGB1 was reversed after ectopic overexpression of Rac1. Moreover, Rac1 overexpression activated production of inflammatory factors in PASMCs treated with Exo-ITGB1 (Fig. 6F and G). The results suggested that miR-429-3p regulated hypoxia-induced PASMCs proliferation, migration and inflammation by targeting Rac1.

### In vivo effects of Exo-ITGB1

**Fig. 7:**
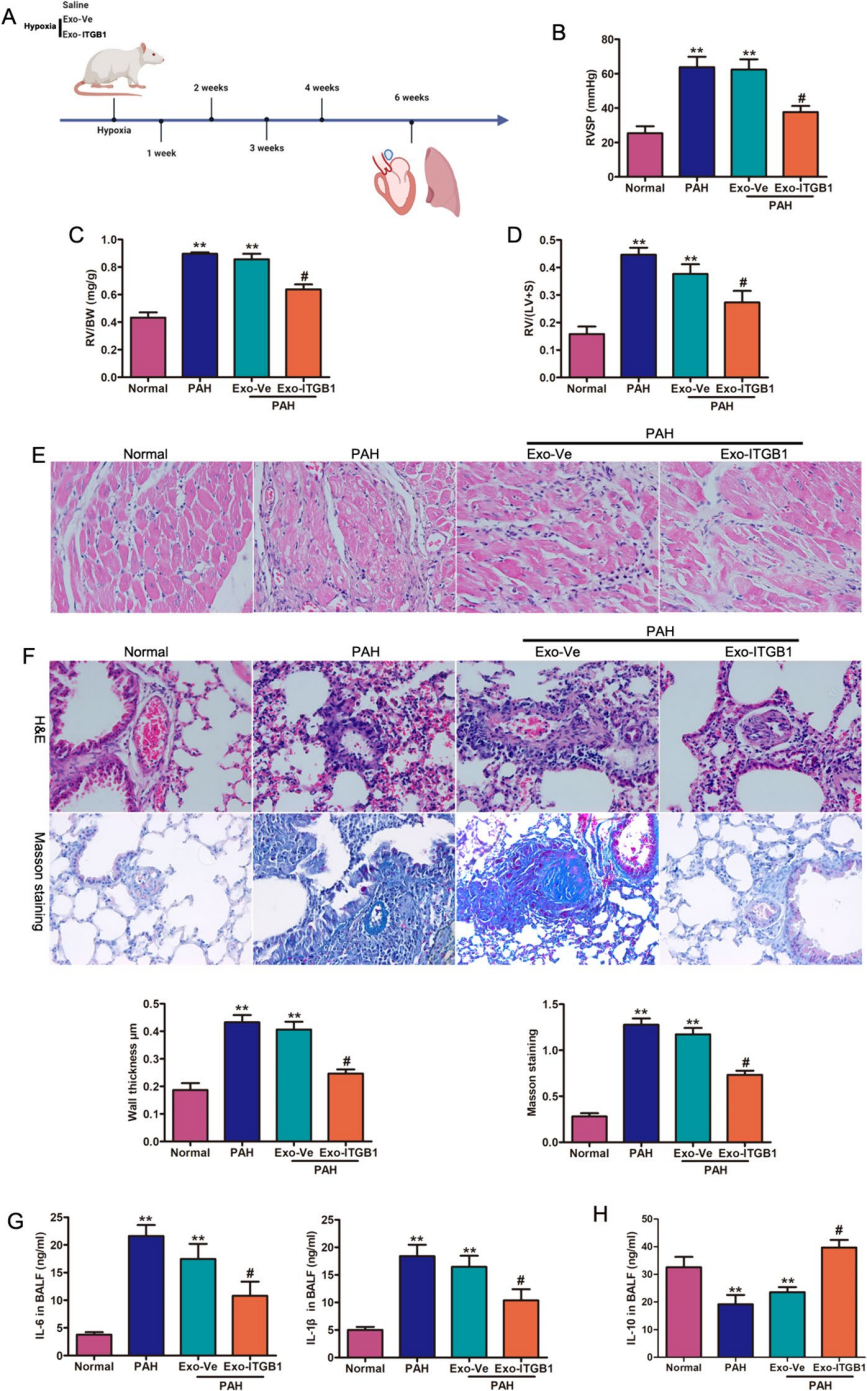
Exo-ITGB1 reverses pulmonary vascular remodeling and right ventricular hypertrophy in PAH mice. (**A**) Schematic illustration showing the in vivo model. Mice were intratracheally instilled with saline (21% oxygen), hypoxia (10% oxygen), hypoxia (10% oxygen) with Exo-Ve and hypoxia (10% oxygen) with Exo-ITGB1 treatment for 4 weeks, and then subjected to histological analysis and hemodynamic measurement. Comparison of RVSP (**B**), RV/BW (**C**), RV/ (LV + S) (**D**) in each group. (**E**) Representative images of H&E staining of heart tissue samples from four different experimental groups. (**F**) Images showing representative H&E and Masson trichrome staining of lung tissue samples from four different experimental groups. Scale bar = 100 μm. Quantitative analysis of the percentage of the pulmonary arterial wall thickness and lung fibrosis. The alteration of inflammatory cytokines IL-6 and IL-1β (**G**), and anti-inflammatory cytokines IL-10 (**H**) in hypoxia-induced PAH. * *p* < 0.05, ** *p* < 0.01 vs. normal group. # *p* < 0.05, Exo-Ve vs. Exo-ITGB1

In hypoxia-induced PAH mice, Exo-miR-429-3p treatment lowered RVSP, RV weight to body weight ratio (RV/BW), and RV to LV + S weight ratio (RV/(LV + S)) (Fig. 7A-D). These effects of Exo-ITGB1 in preventing PAH were further confirmed by histological studies. Histological studies confirmed Exo-ITGB1's prevention of right ventricular hypertrophy and pulmonary artery remodeling (Fig. 7E-F). IL-6 and IL-1β levels in lavage were lower in PAH mice treated with Exo-miR-429-3p compared to controls (Fig. 7G). A significant increase of IL-10 level was observed in PAH mice treated with Exo-miR-429-3p, suggesting Exo-miR-429-3p's therapeutic potential in vascular remodeling and hemodynamic deterioration in hypoxia-induced PAH.

## Discussion

PAH is a complex disease with multiple contributing factors, such as genetic, environmental, and epigenetic factors. Currently available therapies for PAH mainly focus on managing symptoms and improving quality of life. However, there is no known cure for PAH, and the existing treatments only offer partial relief (Forfia et al. 2023; Hoeper 2023). Despite advancements in understanding of PAH, the exact molecular and cellular mechanisms underlying its development and progression are not fully elucidated (Ruopp and Cockrill 2022). Increasing evidence suggested that TCs have shown promise as an alternative to traditional cell therapies for organ tissue injury and repair though directly regulating the impact of information exchange on interstitial behaviors between cells (Cretoiu et al. 2017; Cretoiu 2022). Our study evaluated the effects of ITGB1 on TCs, revealing a significant increase in miR-429-3p expression in TCs post-ITGB1 overexpression, as well as in ITGB1-modified Telocytes-derived exosomes. Exo-ITGB1 was found to suppress hypoxia-induced pulmonary vascular remodeling, proliferation and migration of PASMCs through Rac1 inhibition, suggesting Exo-ITGB1 enriched with miR-429-3p as a potential PAH therapy. Recent research has shown that TCs contributed to intercellular communication and signal transduction, with the ability to acting on neighboring cells through direct contact or paracrine activity (Sukhacheva et al. 2021; Tang et al. 2023). TCs has been reported to share some common features with Mesenchymal stem cells (MSCs), such as: cellular plasticity, tissue regeneration and repair processes, interactions with extracellular environment and homeostasis (Zheng et al. 2013; Asgarpour et al. 2020). The secretome profile from TCs included cytokines, growth factors, mRNAs, like miRNAs, and other non-coding RNAs epigenetic regulators, considered to participate in intercellular exchange with neighboring cells (Tang et al. 2023; Aleksandrovych et al. 2022; Liao et al. 2021). Studies have shown that manipulating miRNAs can have therapeutic effects in PAH. For example, miR-204 was considered as a potential biomarker for PAH, delivery of synthetic miR-204 through intratracheal nebulization resulted in reduced pulmonary arterial blood pressure and improved histological parameters in an experiment on rats with PAH induced by monocrotaline (MCT) (Meloche et al. 2015; Ruffenach et al. 2016; Ji et al. 2022). In this study, no significant change was observed in TCs after ITGB1 overexpression. In order to explore the possible miRNAs mechanisms involved, DEmiRs were detected in TCs after ITGB1 knockdown and nine miRNAs were found decrease. Among them, miR-429-3p had a significantly larger change in expression and further confirmed that miR-429-3p was higher in ITGB1 overexpressed TCs, as well as in ITGB1 modified Telocytes-derived exosomes.

Exosomes are small extracellular vesicles which were secreted by various cell types, including immune cells, stem cells, and tumor cells (Asgarpour et al. 2020; Meloche et al. 2015; Nowak et al. 2023). Exosomes play a crucial role in cell-to-cell communication by facilitating the transfer of biological molecules between cells. They can transport proteins, lipids, nucleic acids (such as RNA and DNA), and other signaling molecules, allowing for the exchange of information and functional materials between cells in local or distant locations (Tienda-Vazquez et al. 2023). In recent years, miRNAs contained in exosomes have received great attention due to their potential as diagnostic biomarkers and therapeutic agents, as miRNA encapsulation in extracellular vesicles provides protection against RNase degradation and enhances its stability in circulation. In this study, exosomes from TCs were isolated and characterized, revealing a notable increase in miR-429-3p expression levels in Exo-ITGB1, suggesting its role in inhibiting migration, proliferation and inflammation of PASMCs.

The abnormal proliferation, migration and inflammation of PASMCs are hallmark pathological features of PAH (Schermuly et al. 2011; Thenappan et al. 2018a). In the PAH model induced by monocrotaline, pulmonary vascular remodeling occurs before the elevation of pulmonary arterial pressure which suggested the change were firstly occurs at cellular level (Thenappan et al. 2018b). MiR-429 is reported to play an important role in various biological processes and has been a widely studied topic in recent years. Previous research has identified miR-429 as a critical regulator in tumors, with lower expression of miR-429 being associated with shorter survival in osteosarcoma patients (Yang et al. 2020). Furthermore, miR-429 overexpression reversed the heightened viability of H9c2 cells induced by swainsonine and mitigated inflammatory injury (Lv et al. 2020). Exosomes of miR-429 derived from adipose-derived stem cells (ADSCs) improve cartilage damage in osteoarthritis via autophagy by targeting FEZ2 (Meng et al. 2023). MiR-429 downregulated in PAH may be beneficial for the development of pulmonary vascular remodeling, and miR-429 inhibits proliferation of PASMCs by downregulating CaSR, then results in reduced Ca2 + influx (Li et al. 2019). Exo-ITGB1 was shown to reduce these pathological processes in PASMCs induced by hypoxia. In a common PAH model induced by hypoxia, treatment with Exo-ITGB1 inhibited pulmonary vascular remodeling, reduced inflammatory cytokine production, and slowed PAH progression, aligning with in vitro findings.

In addition, we further investigated the role of miR-429-3p by investigating potential target genes. Taking bioinformatical analysis, Rac1 was predicted the direct target gene of miR-429-3p by luciferase report. In fact, more and more evidence suggests that Rac1 is involved in the pathogenesis of PAH. Rac1 plays a crucial role in proliferation and migration of PASMCs. Activation of Rac1 promotes PASMCs proliferation and migration, contributing to the vascular remodeling of pulmonary arterial walls observed in PAH (Yu et al. 2012). Activation of Rac1 can stimulate the production of ROS, leading to oxidative damage and the release of pro-inflammatory cytokines and chemokines in the pulmonary vasculature (Dilasser et al. 2022). In our present manuscript, Exo-ITGB1 leads to a significant decrease of Rac1 and overexpression of Rac1 could effectively abolished the inhibition by Exo-ITGB1.

## Conclusions

**Fig. 8:**
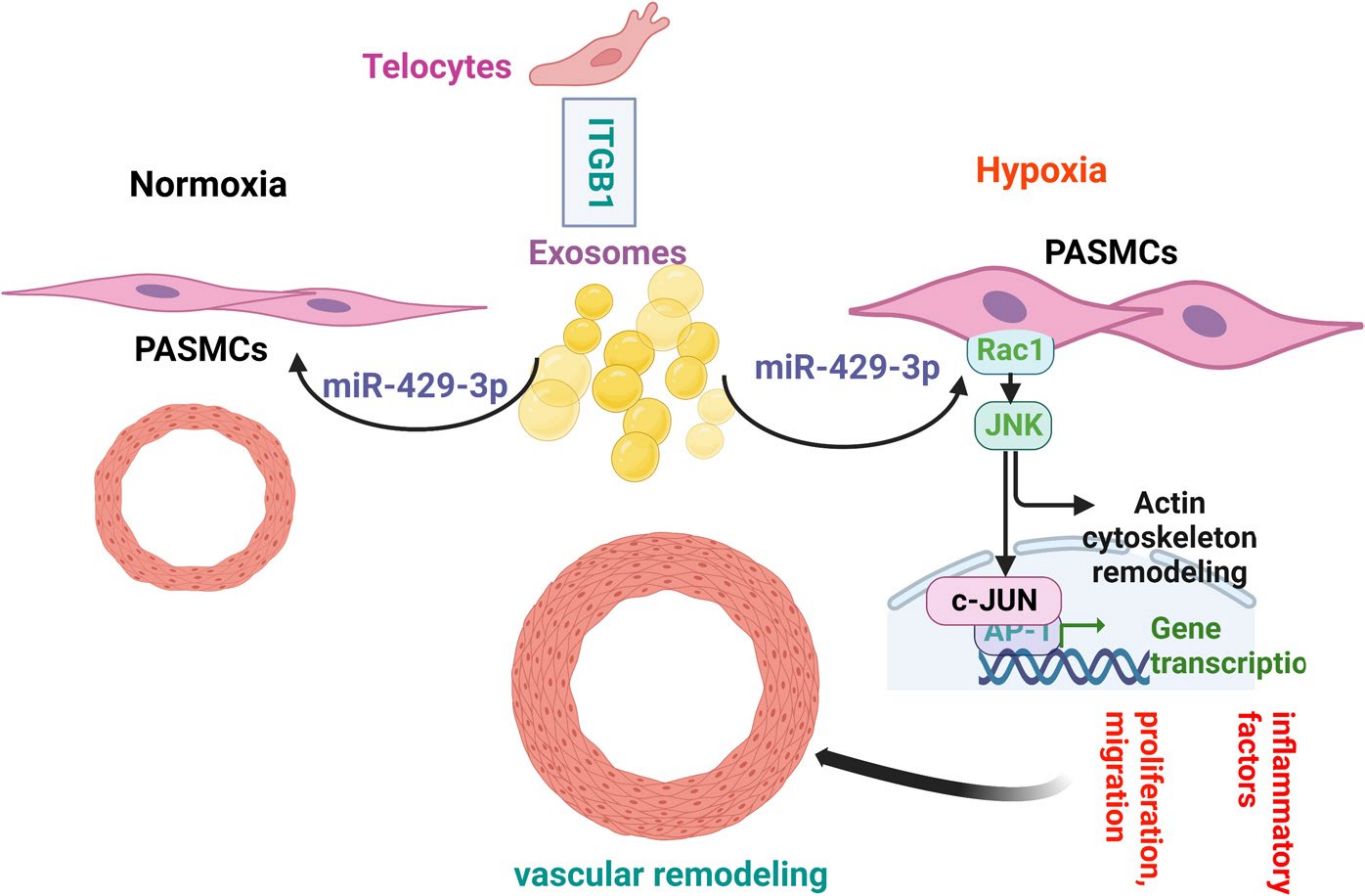
Proposed model illustrating exosomes derived from ITGB1 modified TCs protects hypoxia induced PAH by mediating miR-429-3p/Rac1 axis

In conclusion, this study presents Exo-ITGB1 enriched with miR-429-3p as a novel therapeutic approach for PAH. The results indicate that miR-429-3p, significantly increased in ITGB1-overexpressed TCs and ITGB1-modified Telocytes-derived exosomes, impacts hypoxia-induced PASMCs and PAH progression through Rac1 inhibition (Fig. 8). Further research is needed to confirm the therapeutic efficacy of Exo-ITGB1 in PAH treatment.

## Supplementary Information

Below is the link to the electronic supplementary material.

Supplementary file1 (TIF 4937 KB)

Supplementary file2 (TIF 2639 KB)

Supplementary file3 (TIF 1362 KB)

## Data Availability

No datasets were generated or analysed during the current study.

## Abbreviations

*PAH*: Pulmonary arterial hypertension
*ITGB1-Exo*: ITGB1-modified TCs derived-exosomes
*DEmiRs*: Differentially expressed miRNAs
*PASMCs*: Pulmonary arterial smooth muscle cells
*MSCs*: Mesenchymal stem cells
*ATII*: Alveolar type II cells
*ECM*: Extracellular matrix
*BSA*: bovine serum albumin
*RVSP Right*: ventricular systolic pressure
