# Supplementary figures and images for: Exosomes enriched by miR-429-3p derived from ITGB1 modified Telocytes alleviates hypoxia-induced pulmonary arterial hypertension through regulating Rac1 expression
Source: Cell Biol Toxicol. 2024 May 20;40(1):32. doi: 10.1007/s10565-024-09879-0 (PMC11106170; doi:10.1007/s10565-024-09879-0)

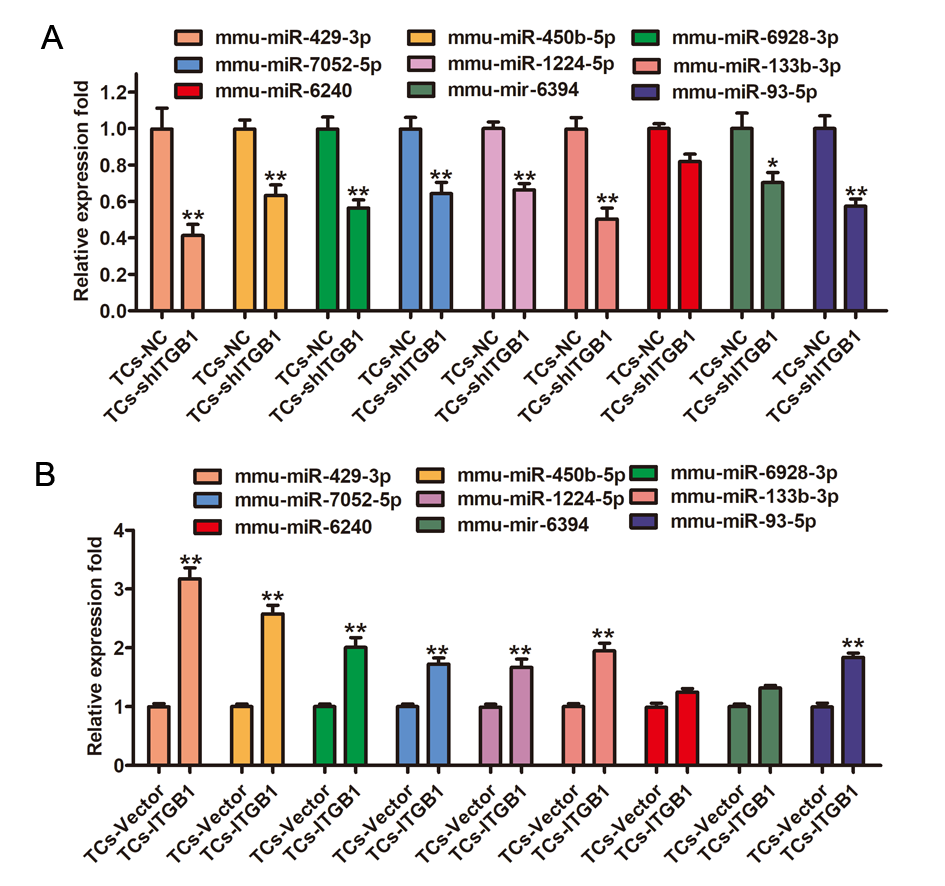

Supplement: Supplementary file 1 — Supplementary file1 (TIF 4937 KB) [file 10565_2024_9879_MOESM1_ESM.tif]

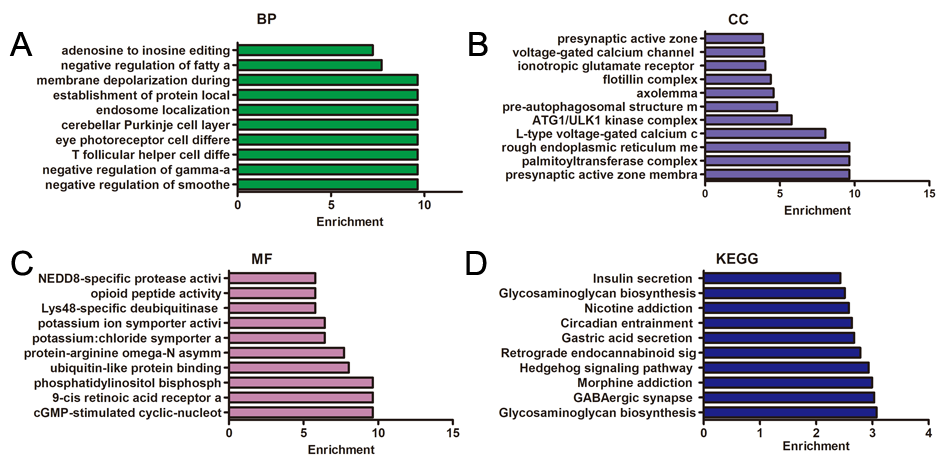

Supplement: Supplementary file 2 — Supplementary file2 (TIF 2639 KB) [file 10565_2024_9879_MOESM2_ESM.tif]

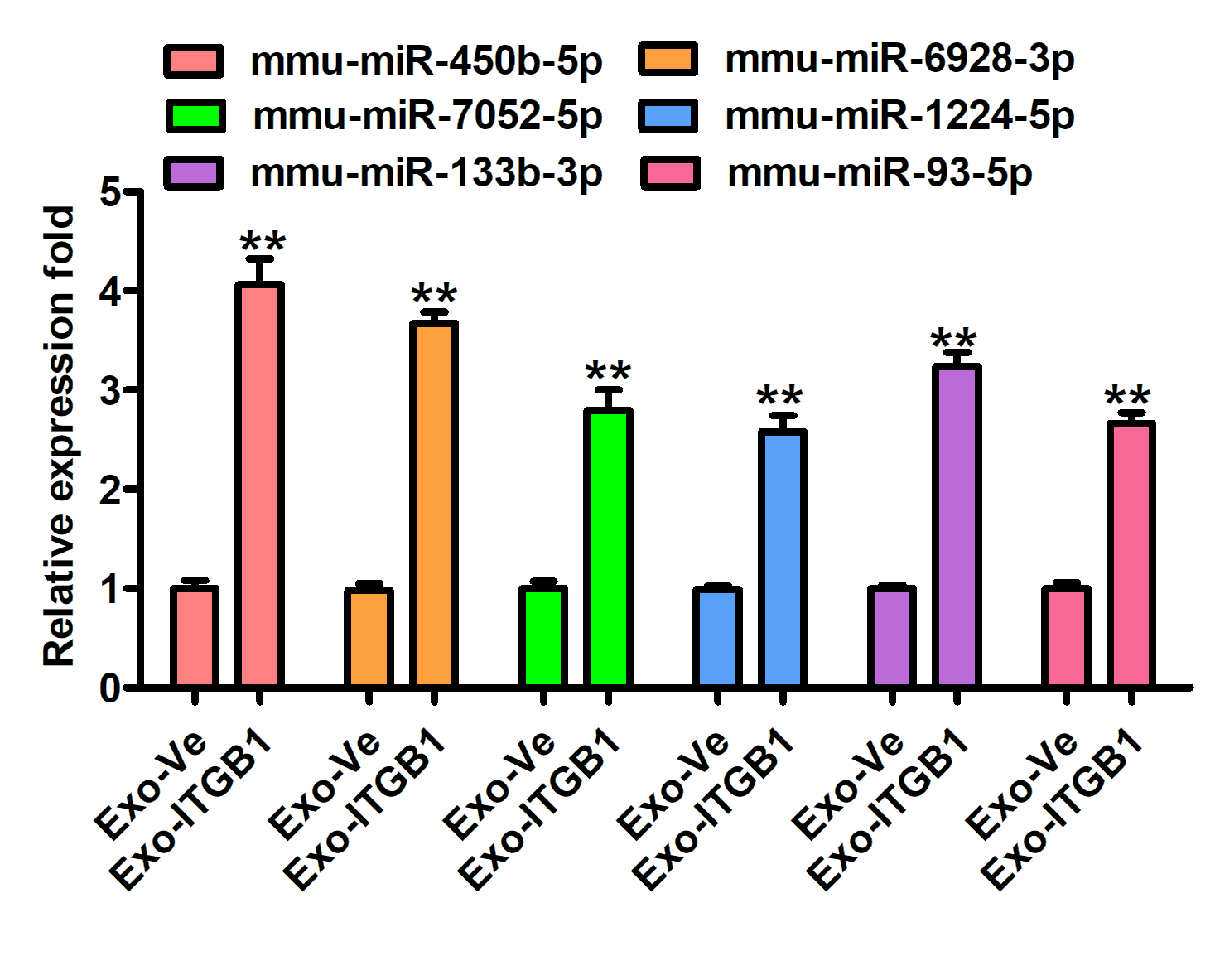

Supplement: Supplementary file 3 — Supplementary file3 (TIF 1362 KB) [file 10565_2024_9879_MOESM3_ESM.tif]
